# Does oral exposure to cadmium and lead mediate susceptibility to colitis? The dark-and-bright sides of heavy metals in gut ecology

**DOI:** 10.1038/srep19200

**Published:** 2016-01-11

**Authors:** Jérôme Breton, Catherine Daniel, Cécile Vignal, Mathilde Body-Malapel, Anne Garat, Coline Plé, Benoît Foligné

**Affiliations:** 1 Univ. Lille, CNRS, Inserm, CHU Lille, Institut Pasteur de Lille, U1019 - UMR 8204 - CIIL - Centre d’Infection et d’Immunité de Lille, F-59000 Lille, France; 2 Univ Lille, Inserm, CHRU Lille, Lille Inflammation Research International Center, U995, Lille, France; 3 Univ. Lille - Center of Biology and Pathology, Toxicology, CHRU of Lille and EA4483, Lille, France

## Abstract

Although the heavy metals cadmium (Cd) and lead (Pb) are known environmental health concerns, their long-term impacts on gut ecology and susceptibility to gastrointestinal autoimmune diseases have not been extensively investigated. We sought to determine whether subchronic oral exposure to Cd or Pb is a risk factor for the development and progression of inflammatory bowel disease (IBD). Mice were exposed to various doses of CdCl_2_ or PbCl_2_ in drinking water for 1, 4 or 6 weeks prior to infection with *Salmonella*, the induction of colitis with dextran sodium sulfate (DSS) or trinitrobenzene sulfonic acid (TNBS). In human cell-based models, exposure to Cd and Pb is associated with reduced transepithelial electric resistance and changes in bacteria-induced cytokine responses. Although 1- and 6-week exposures did not have clear effects on the response to *Salmonella* infectious challenges, 1-week short-term treatments with CdCl_2_ tended to enhance intestinal inflammation in mice. Unexpectedly, subchronic exposure to Cd and (to a lesser extent) Pb significantly mitigated some of the symptoms of DSS-induced colitis and reduced the severity of TNBS colitis in a dose-dependent manner. The possible adaptive and immunosuppressive mechanisms by which heavy metals might reduce intestinal inflammation are explored and discussed.

Genome-wide association studies have highlighted a huge number of susceptibility genes linked to chronic immune diseases, such inflammatory bowel disease (IBD). However, epidemiological studies have shown that the worldwide increase in the incidence of Crohn’s disease (CD) and ulcerative colitis (UC) over the last few decades is mainly due to environmental factors^1^. Thus, the etiology and pathogenesis of IBD are clearly related to changes in lifestyle, diet and pollution levels. This is probably the case in late-onset IBD (i.e. diagnosed in adulthood), associated with more exposure time elapsed^2^. Rappaport and Smith and Lioy and Rappaport^3^,^4^ have extended the initial concept of the individual “exposome” to the whole set of chemicals that enter the body. This encompasses many exogenous factors, such as the diet, dietary pollutants, xenobiotics and endogenous influencing features (e.g. oxidative stress, inflammation) and the microbiome. These factors interact and change over time, and so it is difficult to estimate the causality of environmental signatures. However, it is important to determine the levels of exposure that are likely to tune the many integrated biologic effects (particularly in IBD) and the extent to which putative risk factors might interfere and interact with the host’s adaptive processes.

Only a few clear environmental risk factors for IBD have been identified to date (smoking, appendectomy and early antibiotic use), although discrepancies (and even opposing effects) have been reported as a function of age and the disease subtype^5^,^6^. For example, smoking is positively correlated with the incidence of CD but negatively correlated with the incidence of UC - suggesting that the net effects of myriads of chemicals in cigarette smoke are complex and hard to predict. Indeed, the generation of reactive oxygen species and cadmium (Cd) accumulation may differentially alter the interplay between immune function, oxidative stress and microbiota function in the body as a whole an in the gut in particular. The direct impact of environmental pollutants on gut ecology and subsequent intestinal inflammatory events has not been extensively addressed. It has been shown that the xenobiotic perfluorooctanoic acid was associated with UC in highly exposed workers, although one cannot rule out the possibility whereby the inflammatory state in UC subjects is responsible for increased intestinal absorption of the molecule^7^. It has also been suggested that aluminum is a potent environmental factor for CD induction^8^. The metal’s harmful effects on gut homeostasis have been well established using several *in vitro* and murine models of colitis^9^.

Lead (Pb) and Cd are two widespread, non-essential, heavy-metal pollutants of environmental health concern in both industrialized and developing countries^10^,^11^. Humans can be exposed to Cd and/or Pb from a variety of sources resulting from past and ongoing anthropogenic activities (industrial emissions, car exhaust fumes, fossil fuel combustion, metallurgy and sea pollution). In particular, smoking and dietary intake (through contaminated cereals, fish, shellfish and drinking water) are the major sources of Cd/Pb entry into the gastrointestinal tract^12^. It is also noteworthy that a large proportion of inhaled Cd ends up in the gastrointestinal tract as a result of mucociliary clearance^13^. Heavy metal exposure is harmful because of acute poisoning on one hand and long-term toxicity (due to accumulation) on the other. However, little is known about the impact of subchronic exposure to Cd and Pb on susceptibility to colitis. We therefore sought to determine whether these two heavy metals are potential risk factors for the development and progression of IBD.

In previous works, we characterized the impact of oral exposure to Cd and Pb salts on basal gut physiology and observed (i) time- and dose-dependent dissemination of ingested metals through the blood and target organs, (ii) specific transcriptional signatures in the small intestine and colon and (iii) a clear impact of these metals on intestinal microbial communities^14^,^15^,^16^. However, the complexity of the various toxicological and other endpoints and markers (concomitant anemia, coexisting pro- and anti-inflammatory mediators, oxidative markers, and primary immunosuppressive and possible counter-regulatory adaptive responses) prevented us from predicting or speculating on the heavy metals’ respective effects on gut vulnerability and subsequent intestinal immune responses. After having studied some of these aspects *in vitro* in human-cell-based models, we designed the present study in order to evaluate the influence of oral exposure to Cd and Pb on susceptibility to gut inflammation in a model of *Salmonella typhimurium* infection and two different murine models of colitis.

## Results

### The impact of Cd and Pb on the integrity of a human enterocyte monolayer.

**Figure 1:**
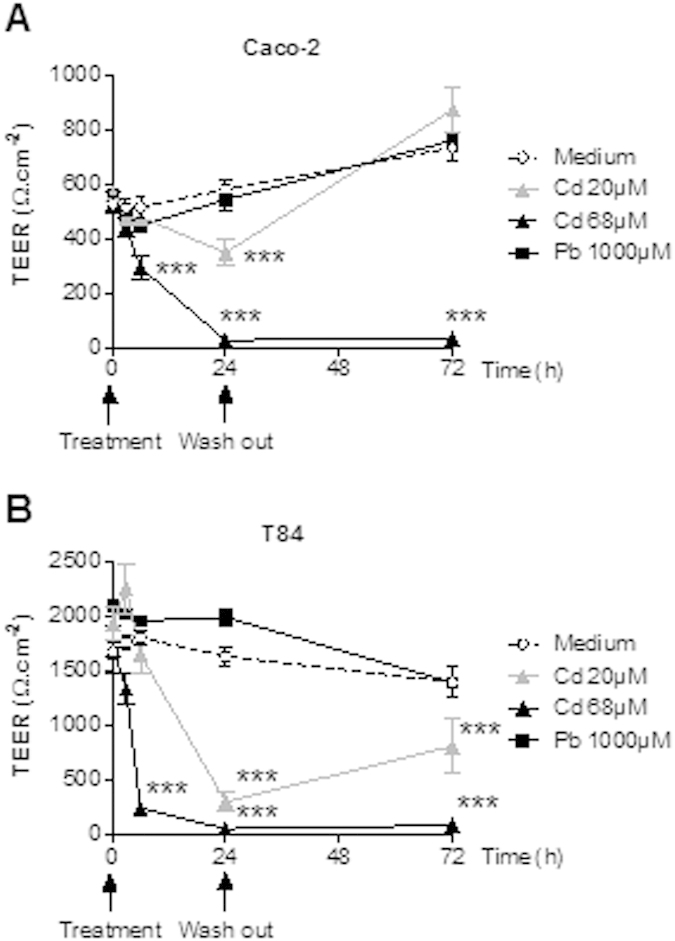
Impact of CdCl_2_ or PbCl_2_ on epithelial layer integrity. Transepithelial electric resistance (TEER) was measured for differentiated Caco-2 (**A)** and T84 (**B**) cells exposed for 24 hours, washed thoroughly and then monitored for a further 48 hours. Data are expressed as the mean ± SEM, n = 3; ***p < 0.001.

Although the Caco-2 cell line is derived from a human colon cancer, it shows certain traits of small intestinal cells. In contrast, the T84 cell line is characteristic of human colonocytes. In order to examine the possible impact of each heavy metal on gut functionality, subtoxic doses of CdCl_2_ or PbCl_2_ were applied to the apical compartment of these two differentiated human intestinal epithelial cell lines. Barrier integrity was evaluated by monitoring the TEER for 24 h and then (after thorough washing) for a further 48 h. Figure 1A,B show that treatment with Cd salts altered the TEER in a concentration-dependent manner for both cell lines, with the higher dose fully abolishing the resistance. After removal of the metal by washing, monolayers exposed to the lower dose rapidly recovered and returned to baseline TEER values (and slightly exceeded for Caco-2, in fact), whereas monolayers exposed to the higher dose never recovered. In contrast, Pb did not appear to affect the TEER, suggesting that Pb does not mediate epithelial damage. Taken as a whole, these *in vitro* data demonstrate that heavy metals have element-specific, time- and potentially reversible dose-related effects on monolayer permeability.

### The impact of Cd and Pb on human immune cell responses

**Figure 2:**
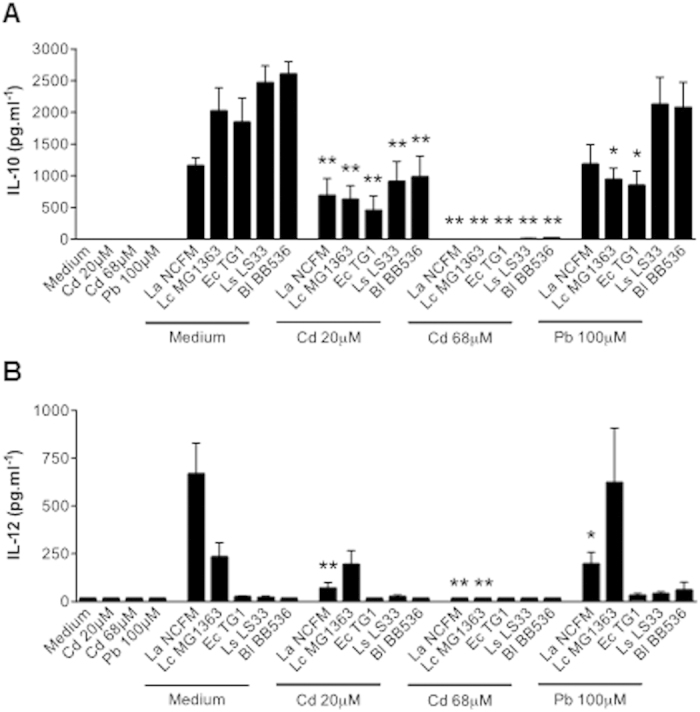
Effect of CdCl_2_ or PbCl_2_ on cytokine release by bacteria-stimulated human Peripheral blood mononuclear cells (PBMCs). Supernatant levels of IL-10 (**A**) and IL-12 (**B**) released by bacteria-stimulated and control human PBMCs in the presence and absence of CdCl_2_ (20 and 68 μM) or PbCl_2_ (100 μM) for 24 hours. A set of bacteria Data are expressed as the mean ± SEM pg.mL^−1^, n = 4 donors; *p < 0.05, **p < 0.01. Metals were simultaneously added to PBMCs with respectively five bacterial strains, a set of microorganisms known for their immune stimulatory properties at various ranges of cytokine levels (*Lactobacillus acidophilus* NCFM, *Lactococcus lactis* MG1363, *Escherichia coli* TG1, *Lactobacillus salivarius* LS33 and *Bifidobacterium longum* BB536)^55^. While heavy metal salts do not induce any pro- or anti-inflammatory cytokines at baseline, they can dose-dependently affect immune responses and cytokine levels when other external stimuli occur simultaneously.

The immunotoxic properties (including both immunosuppression and immunostimulation) of heavy metals have been studied extensively in animal and *in vitro* models. We used human PBMCs to evaluate the immunomodulatory effects (cytokine release) of a 24 h exposure to non-toxic doses of CdCl_2_ and PbCl_2_ in the absence and presence of concomitant bacterial stimulation (Fig. 2A,B). Whereas exposure to the metals alone did not modulate basal cytokine release, concomitant incubation with various types of bacteria was associated with significantly lower levels of IL-10 or IL-12p70 release with 20 μM Cd and the absence of release for 68 μM Cd. Pb (100 μM) has a smaller, less consistent effect on immune responses. These data suggest that heavy metals have potential immunomodulatory effects on immunocompetent cells. However, this type of *in vitro* model cannot fully mimic the integrated physiology of the gut; preclinical models are more reliable for investigating dynamic and adaptive responses.

### The impact of short-term or subchronic exposure to Cd and Pb salts on an oral infectious challenge with *S. typhimurium*

**Figure 3:**
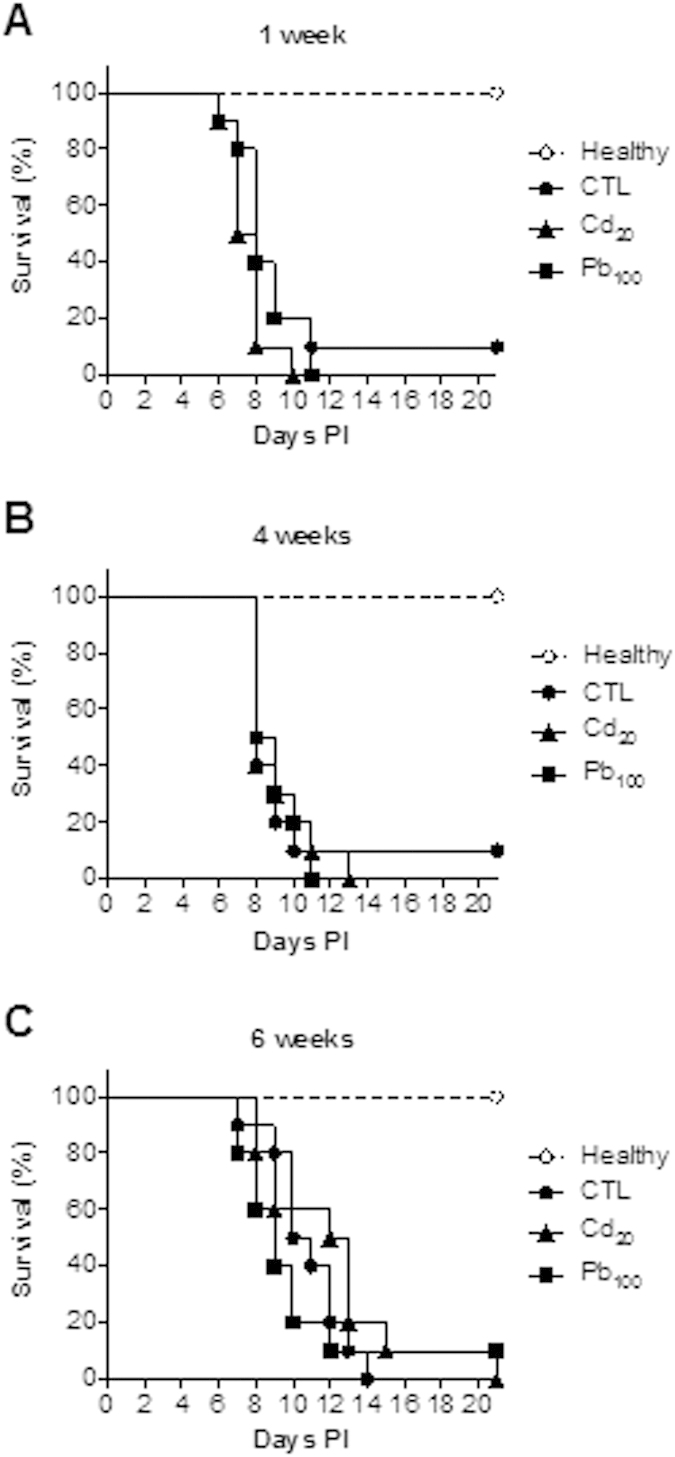
Impact of the CdCl_2_ or PbCl_2_ exposure time on mice survival following an oral *Salmonella* challenge. Survival curves are provided for three weeks post-infection (PI) after 1, 4 and 6 weeks of exposure to Cd or Pb (**A–C**), n = 10 mice per group. Intergroup differences were analyzed in a log rank test.

We first investigated the impact of either short-term ingestion (for 1 week) or subchronic ingestion (for 4 or 6 weeks) of Cd or Pb on the overall innate immune status of the murine intestine by studying survival following an oral challenge with the pathogen *S. typhimurium* mice. Neither Cd (20 ppm in drinking water, i.e. 2.5 mg.d^−1^.kg^−1^) nor Pb (100 ppm, i.e. 12.5 mg.d^−1^.kg^−1^) significantly modified the survival outcomes in any of these situations (Fig. 3A–C, p > 0.1). Thes results show that quite high doses of these heavy metals had a marginal impact on gastrointestinal and systemic antibacterial immune responses

### Short-term exposure to Cd salts accentuates the symptoms of acute DSS- and TNBS-induced colitis

**Figure 4:**
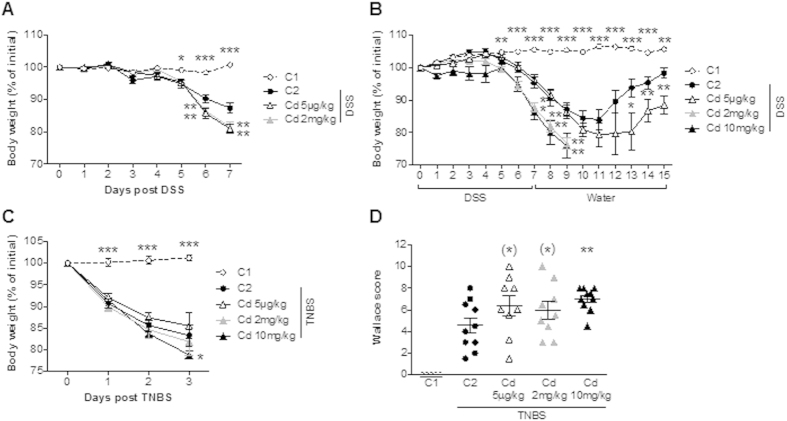
The effect of short-term oral exposure to various doses of CdCl_2_ on the development of chemically-induced colitis in murine models. (**A**) The change in body weight over the course of the DSS experiment. (**B**) The change in body weight recorded over the course of the DSS experiment and after replacement with tap water. Data are quoted as the mean ± SEM, n = 8 mice per condition. (**C**) The change in body weight recorded over the course of the TNBS experiment. (**D**) Macroscopic evaluation of colonic inflammation (the Wallace score); *p < 0.05, **p < 0.01, *0.05 < p < 0.1, compared with C2 (the colitis control).

The main objective of our study was to mimic realistic environmental exposure conditions and characterize the long-term effects of Cd and Pb salts on susceptibility to colitis. We first sought to determine the effect of a short period of heavy metal exposure on body weight loss (a major inflammation-associated sign). In an initial experiment, a week of daily exposure to either very low-dose (5 μg.kg^−1^) or high-dose (2 mg.kg^−1^) CdCl_2_ was associated with weight loss in mice subsequently exposed to DSS-related (p < 0.01 vs. controls, Fig. 4A). We then assessed the effect of very low-dose (5 μg.kg^−1^) or high-doses (2 and 10 mg.kg^−1^) CdCl_2_ in a model of healing following colitis induced by DSS (Fig. 4B). In DSS control animals (C2), DSS interruption led to a recovery phase with a weight regain. In mice exposed to the low dose 5 μg.kg^−1^ Cd, body weight recovery was slower and only partial. Mice exposed to the higher doses kept losing weight after DSS cessation until the ethical defined endpoint (i.e. 20%) was reached, thus prompting us to euthanize the animals at day 9.

When assessing the consequences of 5 days of Cd exposure on the severity of TNBS-induced colitis, only the higher dose (10 mg.kg^−1^) significantly aggravated the body weight loss (p < 0.05, Fig. 4C) and the score for macroscopic colon injury (p < 0.01, Fig. 4D). The differences with lower doses were not significant (0.05 < p < 0.1). Taken as a whole, these data suggest that acute exposure to Cd had a noticeable impact by exacerbating inflammation and delaying remodeling and tissue repair pathways.

### Subchronic exposure to Cd and Pb salts does not have a marked effect on DSS-induced colitis

**Figure 5:**
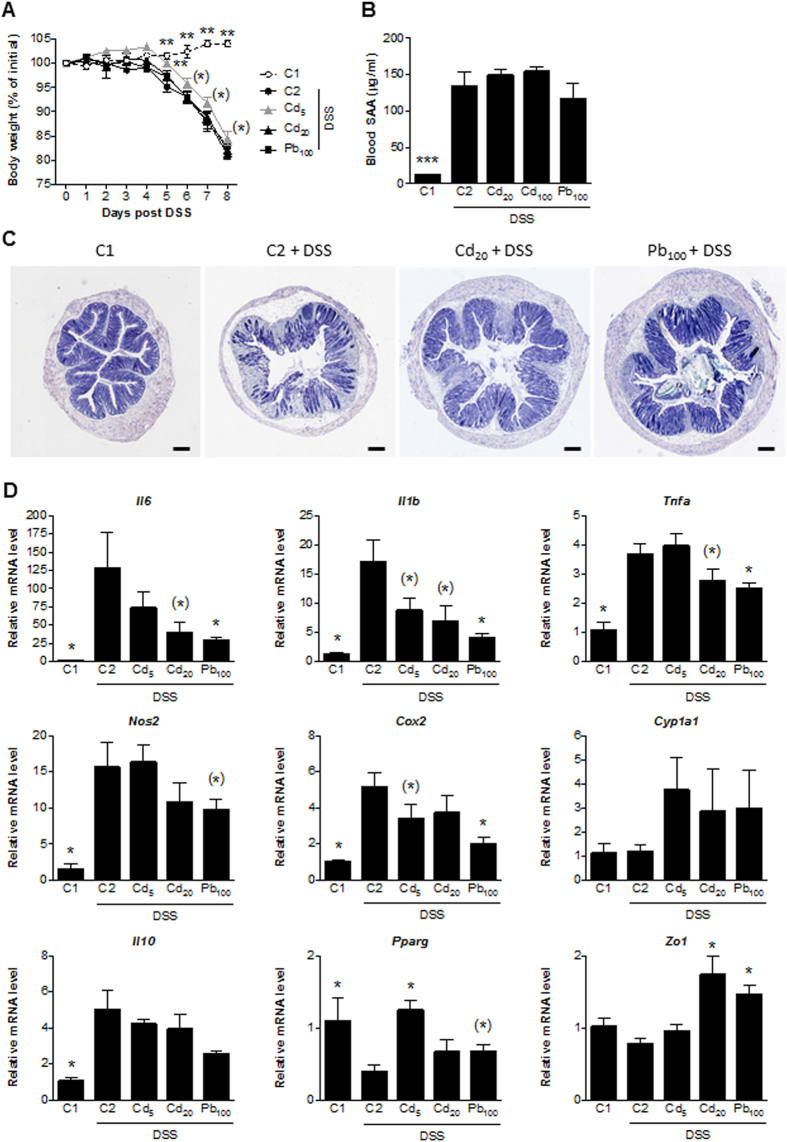
Impact of 6 weeks of oral exposure to CdCl_2_ or PbCl_2_ on DSS-induced colitis in mice. (**A**) The mean change in body weight over the course of the experiment. (**B**) Serum levels of SAA protein (in μg.mL^−1^). (**C**) Representative photomicrographs of the histological features of May-Grünwald-Giemsa-stained colon sections (bar = 200 μm). (**D**) mRNA expression levels of specific genes in the colon, relative to that of *Actb*. Data are quoted as the mean ± SEM, n = 8 mice per condition; *p < 0.05, **p < 0.01, ***p < 0.001, *0.05 < p < 0.1, compared with C2 (the colitis control).

Ingestion of CdCl_2_ (5 and 20 ppm, corresponding respectively to 0.5 and 2.5 mg.d^−1^.kg^−1^) or PbCl_2_ (100 ppm, i.e. 12.5 mg.d^−1^.kg^−1^) in drinking water for 6 weeks did not significantly impact the main clinical outcomes in mice with DSS-induced colitis, such as body weight loss (Fig. 5A) and SAA protein levels (Fig. 5B). However, despite the absence of obvious changes in the signs of disease, the mice’s external appearance (e.g. the sheen of the fur) and behavior (e.g. searching activity) were clearly better in the metal-exposed groups. The Cd- and Pb-associated relief of colitis symptoms was confirmed histologically, with less epithelial damage in the metal-exposed groups (Fig. 5C). Accordingly, we observed lower transcriptional activity of pro-inflammatory genes (such *Il6*, *Il-*β, *Tnfa, Nos2* and *Cox2*) in colon biopsies (Fig. 5D); this suggests that both metals had a clear, positive influence on components of the local inflammatory response. Interestingly, mRNA expression of IL-10 (a regulatory, adaptive cytokine often induced during inflammatory events) was not modified by exposure to heavy metal salts. mRNA expression of the *Pparg* and *Zo-1* genes was unaffected or slightly induced, depending on the dose of heavy metal.

### Subchronic exposure to Cd and Pb salts has protective effects in TNBS-induced colitis.

**Figure 6:**
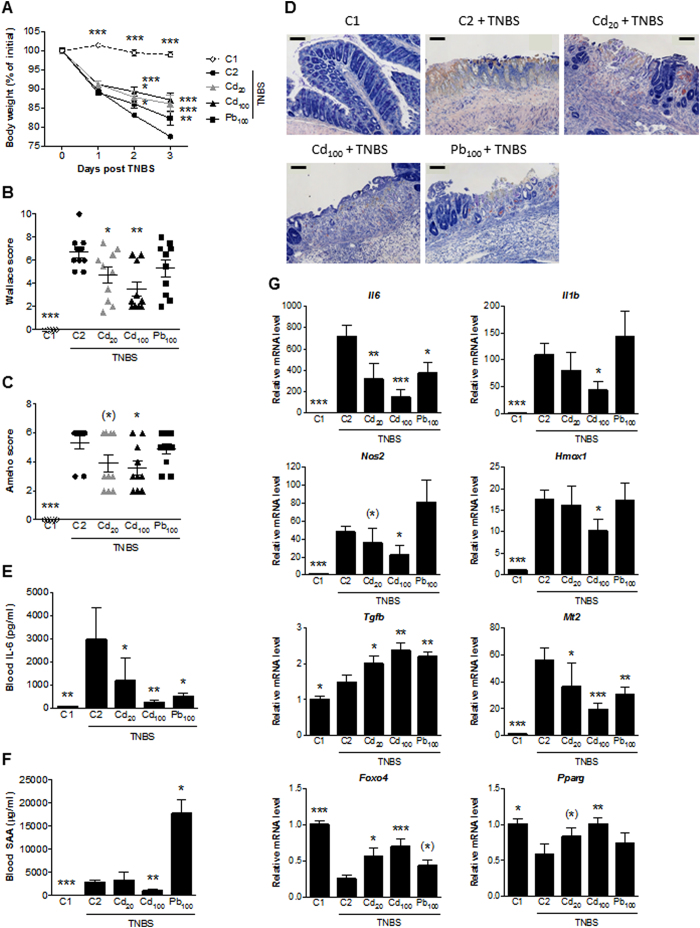
Impact of 6 weeks of oral exposure to CdCl_2_ or PbCl_2_ on TNBS-induced colitis in mice. (**A**) The mean change in body weight over the course of the experiment. (**B**) Macroscopic evaluation of colonic inflammation (the Wallace score). (**C**) Histopathological evaluation of colonic inflammation (the Ameho score). (**D**) Representative photomicrographs of histological features of May-Grünwald-Giemsa-stained colon sections (bar: 100 μm). (**E,F**) Serum levels of IL-6 and SAA protein. (**G**) mRNA expression levels of specific genes in the colon, relative to that of *Actb*. Data are quoted as the mean ± SEM, n = 10 mice per condition; *p < 0.05, **p < 0.01, ***p < 0.001, *0.05 < p < 0.1, compared with C2 (the colitis control).

Six weeks of oral exposure of CdCl_2_ (20 ppm and 100 ppm) and PbCl_2_ (100 ppm) in drinking water was associated with significantly lower TNBS-induced colonic damage in terms of body weight loss (Fig. 6A), macroscopic lesion (Fig. 6B), histological scores (Fig. 6C) and necrosis and epithelial loss (Fig. 6D). Levels of general markers of inflammation were also drastically lower following long-term exposure to Cd and/or Pb (Fig. 6E,F). These observations were consistent with the transcriptional signatures in inflamed colons (Fig. 6G) (although dependent on the doses and the metal in question). Indeed, the TNBS-induced upregulation of inflammatory genes (such as the immune-related *Il6*, *Il1b* and *Tnfa* and oxidative-stress-related genes (such as *Nos2* and *Cox2*) was less marked or down-regulated in metal-exposed groups. We also observed greater induction of transcripts of *Foxo4*, an endogenous inhibitor of NfκB^17^ that is downregulated by TNBS. The expression of certain genes related to remodeling and anti-inflammatory or anti-oxidant functions are sometimes actively induced in an inflammatory context and thus reflect the adaptive response of the mucosa. This is the case for *Tgfb* (which was upregulated by both Cd and Pb), whereas *Il10, Hmox1* and the metallothioneins (MTs) *Mt1* and *Mt2* were less expressed in metal-exposed animals with inflammation.

## Discussion

It is well known that both short-term and chronic oral exposure to heavy metals can induce (i) breaches in the gut barrier, (ii) local and systemic immunosuppressive effects and (iii) the genesis of a pro-oxidant environment. This context may contribute to create “the perfect storm” to favor chronic intestinal disease. However, our present results show that subchronic exposure to Cd or Pb salts in drinking water may have a beneficial effect in mice by mediating protection against experimentally-induced acute colitis. This phenomenon may involve several factors inside and outside the intestine.

At baseline (without colitis), it is known that Cd induces substantial levels of MTs in all parts of the small intestine and in the colon^15^, but this cannot be sufficient to explain all the anti-inflammatory effects (in terms of threshold or time of impregnation). We thus hypothesized that adaptive processes are also involved. Based on our previous experiments^15^, we can state that no induction of SAA neither IL-6 was induced *per se* by the metals and thus, we cannot claim that Cd or Pb salts are pro-inflammatory.

We first confirmed that Cd (but not Pb) altered the TEER in an *in vitro* reconstituted epithelium system. Our findings also suggest that Cd might be associated with a reversible reduction in epithelial permeability *in vivo*. Furthermore, Cd and Pb may lower immune responsiveness because both heavy metals reduced bacteria-elicited cytokine release (but not baseline release). Given that pro-and anti-inflammatory pathways were affected in similar ways, it is hard to predict whether the inflammatory response will be exacerbated or dampened. However, Cd- and Pb-exposed mice were no more susceptible than controls to *S. typhimurium*, suggesting that the metals only have a moderate impact on intestinal innate immunity *in vivo*. Nevertheless, these harmful effects may account for the exacerbation of experimentally induced colitis observed in mice after short-term exposure. The alleviation of colitis after longer, continuous exposure to Cd and Pb may involve other mechanisms.

Maintaining homeostasis of the intestinal mucosa involves adaptive, counter-regulatory processes that might be preferentially induced by longer exposure to toxic heavy metals. Boirivant *et al.*^18^ showed that breaches in the intestinal barrier (caused by ethanol and another tight junction disrupter) are necessary for efficient, protective homeostatic regulatory T-cell responses to mucosal inflammation. Likewise, it has been reported that superoxide and NO have divergent roles in intestinal inflammation^19^, and that physiologic reactive oxygen species (ROS) signaling regulates homeostatic processes^20^. It has also be established that exogenous bacteria can prevent intestinal NF-kB activation by increasing epithelial levels of ROS^21^, whereas lactobacilli use H_2_O_2_ to activate PPARg in epithelial cells and thus modulate inflammation^22^. Although massive oxidative stress contributes to failure of the intestinal barrier, a moderate increase in intracellular ROS concentrations paradoxically affords protection and prevents intestinal barrier dysfunction via the upregulation of oxidative defense mechanisms^23^. We recently investigated the impact of subchronic Pb and Cd exposure on gut ecology, with a view to developing a more comprehensive view of the consequences of environmental exposure at baseline (i.e. before the induction of colitis)^14^,^15^,^16^. We found that the mRNA expression of inducible enzymes coded by genes such as *Nos2* and *Gpx2* did not change significantly (or was even somewhat lower) in the colon of metal-exposed mice. In contrast, transcription of *Mt1* and *Mt2* in the duodenum and the colon was upregulated. Furthermore, a significant, consistent rise in the levels of *Cyp1a1* mRNA was measured in all parts of the intestine of exposed animals, and *Hmox1* was clearly induced in the distal ileum. All of these genes (either separately or conjointly) may contribute to increased levels of anti-oxidants and may thus further limit colitis. Heavy-metal-induced genes have multiple functions, and - at least in the gut - chronic contamination by xenobiotics may paradoxically exert some beneficial or protective features. Indeed, the MTs are known to exert anti-inflammatory effects both inside the gut^24^,^25^ and outside the gut^26^,^27^ and to protect against rheumatoid arthritis and colitis^28^. Several mechanisms are involved, including oxygen radical scavenging and immunomodulation. However, the MTs’ role during disease onset and progression in animal models of colitis and IBD is not yet clear^29^. Similarly, activation of the aryl hydrocarbon receptor (AhR) pathway (induced in colitis, along with *Cyp1a1*) ameliorates DSS-induced colitis in mice^30^,^31^. Other examples of the modulation of mucosal immune responses by environmental factors involve the AhR and the estrogen receptor^32^,^33^; this reinforces evidence in favor of an estrogen-like role of Cd in the intestine^34^ and related anti-inflammatory role in the gut^35^. There are abundant literature data on the protective role of *Hmox1* in clinical and experimental colitis^36^,^37^,^38^ and mechanisms might also involve Cd-induced protective autophagy^39^. In the context of the present study, specific gene-by-gene inactivation could be used to determine the respective roles in Cd-mediated protection but one can legitimately hypothesize that several genes act together.

The low levels of colonic and blood iron associated with the chronic ingestion of Cd^14^ might also partly explain the Cd-associated alleviation of colitis – perhaps via a drop in oxidative stress and other indirect mechanisms^40^,^41^.

Lastly, specific modification of the gut microbiota might also account for how heavy metals can protect against or promote colitis^1^,^9^,^42^. Our previous studies have shown that Cd and Pb can affect the structure of microbiome and change the bacterial community, leading among other things to a lower proportion of *Lachnospiracea* and a higher proportion of *Turicibacter*^16^. We hypothesize that adaptation of the microbiota to a heavy-metal environment drives the differential responses observed in the present study. For example, *Turicibacter* have been detected in the ileal pouch of a patient with ulcerative colitis^43^ and in human appendicitis^44^, whereas low levels of this genus were observed in dogs with idiopathic IBD^45^. Interestingly, high levels of *Turicibacter* were observed in colitis-resistant CD8 knock-out mice, where the genus is potentially involved in the anti-inflammatory phenotype^46^. Furthermore, the fact that IBD is associated with a reduction in the diversity of taxonomic clusters and specific groups (i.e., *Lachnospiraceae*) suggests that bacterial groups might have an important role in gastrointestinal health. However, further research is required to evaluate the functional changes associated with this type of intestinal dysbiosis. Although human IBD and experimental colitis in mice are clearly associated with specific shifts in intestinal microbiota composition, the extent to which these microbiota dynamics are indicative of health or disease is not clear^47^. Oral exposure to Cd and Pb may generate bidirectional, adaptive interactions between the host and the microbiota. Again, we are not yet able to discriminate between causes and causality or to state that inflammation-associated enterotypes or strains can promote or protect against colitis. It remains difficult to differentiate between the effects of isolated host factors, microbial factors and host-microbiota interactions on intestinal homeostasis and toxicological responsiveness.

In line with our present results, Ansari *et al.*^48^ showed that 3 weeks of exposure to low-dose CdCl_2_ (5 ppm) during the initiation phase of a collagen-induced arthritis model in rats reduced disease progression, whereas exposure to a higher dose (50 ppm) exacerbated the severity. This recent work confirms that Cd salts potentially have a collateral, beneficial effect on a chronic inflammatory disease through stimulation of anti-oxidant pathways and downregulation of NfκB and pro-inflammatory cytokine pathways. Beneficial effects for other metals of health concern have already been reported, although they depend on element speciation and administration route. In mice, for example, sodium arsenite was associated with a reduction in the severity of DSS-induced experimental colitis^49^ and arsenic trioxide had anti-inflammatory consequences in TNBS-induced colitis^50^. Likewise, preclinical studies using cobalt^51^, lithium^52^, strontium^53^ and uranium^54^ also found a clear anti-inflammatory potential. Lastly, some preliminary data strongly suggest that mice exposed to methylmercury for 3 weeks are resistant to DSS-associated inflammation (Body-Malapel and Vignal, unpublished data). Taken as a whole, these data reinforce the notion whereby specific doses and durations of exposure to dietary or contaminant heavy metals may variously elicit neutral, pro- or anti-inflammatory phenomena via a number of interrelated pathways.

Lastly, some of the potentially “positive” side effects of heavy metals based on the host’s intestinal adaptome might not underestimate toxicogenic effects at other sites (such as nephrotoxicity) or harmful long-term consequences (such as colon tumorigenesis).

## Conclusion

Our present observations provide insights into the Cd- and Pb-mediated modulation of inflammatory processes and emphasize that clinical outcomes vary considerably as a function of dose and exposure time. This may also apply to other xenobiotics involved in the onset of IBD, especially when the body is exposed to mixtures of metals or other dietary and environmental risk factors. Several factors can influence the subtle pro-/anti-oxidant balance and subsequent immune responses by either disrupting or (as a sort of “collateral benefit”) reinforcing homeostatic mechanisms. Additional epidemiological and toxicological studies are needed to determine the integrated role of the environmental exposome in gut ecotoxicology and thus identify risk factors, key regulators and novel therapeutic approaches in IBD and other immune-related disorders. This research must take account of the host’s complex responses and interplay between (i) the exposome, (ii) the adaptome and (iii) the emerging techniques in microbiome science (including metagenomics and metabolomics), in order to better manage environmental factors for human health.

## Methods

### Materials

Chemicals and reagents were purchased from Sigma-Aldrich Chemical (St Quentin Fallavier, France), unless otherwise stated.

### Cell lines and *in vitro* transepithelial electric resistance (TEER) measurements

Human intestinal epithelial HTB37 (Caco-2) and CCL248 (T84) cells (purchased from the American Type Tissue Collection) were seeded (2.10^5^ cells.cm^−2^) on Transwell polycarbonate cell culture inserts with pore size of 3 μm (Corning Costar) and cultivated for up to 21 days in appropriate media. TEER was measured using the Millicel-ERS epithelial volt-ohmmeter (Millipore) and expressed in Ω.^cm−2^ as the mean of triplicate wells. Subtoxic doses (as previously determined in MTT cell viability assays) of CdCl_2_ (20 μM and 68 μM) and PbCl_2_ (1000 μM) were applied to the apical compartments. TEER was monitored for 24 h and then (after intensive washing of the cells) for a further 48 h.

### *In vitro* immunomodulation assays

Peripheral blood mononuclear cells (PBMCs) were isolated from the blood of four healthy donors as already described^55^. Nontoxic doses of CdCl_2_ (final concentration: 20 or 68 μM) or PbCl_2_ (100 μM) were added in the absence or presence of 10 μl of thawed bacterial suspensions (in 20% glycerol in PBS, with a bacterium-to-cell ratio of approximately 10:1). Cytokine release (human IL-10 and IL-12p70) was measured in an ELISA using pairs of antibodies (BD Pharmingen^TM^). All experimental protocols were approved by our institution committees (INSERM, CNRS and Institut Pasteur de Lille). Blood sampling from healthy informed donors were done upon approved agreement of volunteers (signed consents) by authorized staff in accordance with the abovementioned committees, i.e. (INSERM, CNRS and Institut Pasteur de Lille).

### Animal experiments and ethics statements

Animal experiments were performed in accordance with the guidelines issued by the Institut Pasteur de Lille’s Animal Care and Use Committee, which are based on the Amsterdam Protocol on Animal Protection and Welfare and Directive 86/609/EEC on the Protection of Animals Used for Experimental and Other Scientific Purposes, updated in the Council of Europe’s Appendix A. The animal experiments also complied with French legislation (Government Act 87-848) and the European Communities Amendment of Cruelty to Animals Act 1976. All the studies were approved by the local investigational ethics review board (Nord-Pas-de-Calais CEEA N°75, Lille, France; protocol reference numbers 192009R, 212009R, 222009R and 042011).

Short-term exposure (1 week) was performed by daily oral gavage with 5 μg.kg^−1^, 2 mg.kg^−1^ or 10 mg.kg^−1^ of CdCl_2_. Subchronic exposure consisted of 1, 4 or 6 weeks of *ad libitum* access to contaminated drinking water containing 20 or 100 ppm CdCl_2_ (corresponding to daily exposures of 2.5 and 12.5 mg.kg^−1^, respectively, based on a daily intake of 2.5 ml) or 100 ppm of PbCl_2_ (corresponding to 12.5 mg.kg^−1^ daily). Indeed, we retained these two ranges of optimal doses respectively, as the modulation of inflammation does not require the same intensity between each model, i.e. DSS and TNBS, based on preliminary studies (data not shown).

### *Salmonella typhimurium* challenges

Ten- to twelve-week-old female BALB/c mice were intragastrically challenged with 5 × 10^5^ CFU (200 μL) of an exponential culture of *Salmonella enterica* serovar Typhimurium strain C5^56^ grown in LB broth and resuspended in distilled water. Animals were kept in positive-pressure cabinets, and mortality was monitored daily over a three-week period, as previously described^57^.

### Induction of colitis with dextran sodium sulfate (DSS) and trinitrobenzene sulfonic acid (TNBS)

DSS and TNBS chemically induce colitis models of gut inflammation, sharing common features but also exhibiting specific traits. Since a single model of colitis does not resemble all the features of human IBD, we used two different models, namely trinitrobenzenesulfonic acid (TNBS)- and dextran sulphate sodium (DSS)-induced colitis, to strengthen the value of our findings. Therefore, these two models are thought to be reliable and rather complementary when studying the pathogenesis of IBD, and in this particular case, the overall inflammation inside the gut, as a general deleterious inflammatory context in rodents. Two models of chemically-induced acute colitis were implemented in 12-week-old female BALB/c, as previously described^58^. For the DSS model, mice (n = 8 per group) were exposed to 5% DSS molecular weight 36–50 kDa (MP Biomedicals) in their drinking water for seven consecutive days prior to necropsy. For the TNBS model (n = 10 mice per group), the colitis was triggered by the intrarectal administration of 50 μl TNBS (100 mg.kg^−1^) in 0.9% NaCl/ethanol (50/50 v/v). Three days after the induction of colitis, mice were euthanized and blood samples were collected immediately. The serum was separated and frozen (−20 °C). After dissection, two independent observers blindly scored the macroscopic inflammation of the colon by using the Wallace score^59^. The Wallace score rates macroscopic lesions on a scale from 0 to 10 based on features reflecting inflammation, such as hyperaemia (score 1 to 2), moderate to intense thickening of the bowel (score 2 to 3), and the gradual extent of 1 cm-long ulceration (score 3 to 7), according to the maximal mouse colon length. Dead mice due to over-inflammation were scored at 10, the maximal inflammatory score seen in mouse models. Following examination under microscope, tissue lesions were scored according to the Ameho criteria^60^. Briefly, histological findings identical to those of normal mice were scored as 0, whereas a score of 1 indicated mild mucosal and/or submucosal inflammatory infiltrate and edema, punctate mucosal erosions, and intact muscularis mucosae; the same histological findings involving 50% of the specimen were scored as 2. Prominent inflammatory infiltrate and edema, deeper areas of ulceration extending through the muscularis mucosae into the submucosa, and rare inflammatory cells invading the muscularis propria but without muscle necrosis were scored as 3, the same histological findings involving 50% of the specimen were scored as 4, extensive ulceration with coagulative necrosis with deep extension of the necrosis into the muscularis propria were scored as 5, and the same histological findings involving 50% of the specimen were scored as 6. The Wallace and Ameho scoring systems used here are the most appropriate for the TNBS model; they both report a gradual analysis of the lesions. However, they cannot be used to score the diffuse and patchy lesions occurring in DSS. Indeed, the body weight loss appears for us to be the most relevant clinical marker/symptom of inflammation as previously described^58^ and we decided to retain this main marker as a representative of the pathology.

For both models, distal colon samples (0.5 cm in length) were processed with RNA stabilization solution (RNA-later, Ambion, Life Technologies) and stored at −80 °C for transcriptional analysis. Samples for histologic analysis were processed for paraffin-embedding, prepared as 5 μm sections and stained with May Grünwald-Giemsa reagent to score tissue lesions according to the Ameho criteria. Blood levels of murine IL-6 and serum amyloid A (SAA) protein were measured using ELISA kits (BD Pharmingen) and (Biosource International) respectively.

### Quantitative reverse-transcriptase polymerase chain reaction (RT-PCR)

Samples were homogenized using the FastPrep instrument (MP Biomedicals), total RNA was isolated using RNAspin columns (Macherey-Nagel). Reverse transcription and real-time PCR were performed with reaction kits (the High-Capacity cDNA RT Kit) and reagents (Universal PCR Master Mix, Applied Biosystems), according to the manufacturer’s instructions. PCR reactions were performed with a MX3005P machine (Stratagene, Agilent Technologies). A custom gene expression assay (TaqMan, Applied Biosystems) was used with commercially designed and validated primers (Table S1). The housekeeping gene beta actin was run as an internal control. Data were analyzed using the 2^−ΔΔCt^ method and expressed as a fold-increase over the control group’s values.

### Statistical analysis

All analyses were performed by comparing experimental groups with their respective controls in a non-parametric, one–way analysis of variance (the Mann–Whitney *U*-test) or a two-tailed Student’s-*t-*test, as appropriate (version 6.0, GraphPad Software Inc.). Survival rates after *Salmonella* challenge were analyzed using the log rank test. Data are presented as the mean ± SEM. Differences were judged to be statistically significant when the p-value was <0.05. However, p-values between 0.05 and 0.1 are specified to indicate trends.

## Additional Information

**How to cite this article**: Breton, J. *et al.* Does oral exposure to cadmium and lead mediate susceptibility to colitis? The dark-and-bright sides of heavy metals in gut ecology. *Sci. Rep.* **6**, 19200; doi: 10.1038/srep19200 (2016).

## Supplementary Material

Supplementary Information
