# Supplementary material for: Does oral exposure to cadmium and lead mediate susceptibility to colitis? The dark-and-bright sides of heavy metals in gut ecology
Source: Sci Rep. 2016 Jan 11;6:19200. doi: 10.1038/srep19200 (PMC4707487; doi:10.1038/srep19200)
Supplement: Supplementary Information [file srep19200-s1.pdf]

Does oral exposure to cadmium and lead mediate susceptibility to colitis? The dark-and-bright sides of heavy metals in gut ecology

Jérôme Breton, Catherine Daniel, Cécile Vignal, Mathilde Body-Malapel, Anne Garat, Coline Plé, Benoît Foligné

Table S1: target genes and the corresponding primer accession numbers

Gene name	Abbreviation	Catalog number
Actin beta	*Actb*	Mm 01205647_g1
Metallothionein 1	*Mt1*	Mm 00496660_g1
Metallothionein 2	*Mt2*	Mm 00809556_s1
Nitric oxide synthase 2 (inducible)	*Nos2*	Mm 00440502_m1
Superoxide dismutase 1	*Sod1*	Mm 01344233_g1
Glutathione peroxidase 2	*Gpx2*	Mm 00850074_g1
Heme oxidase 1	*Hmox1*	Mm 00516004_m1
Transforming growth factor, beta 1	*Tgfb1*	Mm 00441724_m1
Cyt P450-fam.1 subfam.A, polypep 1	*Cyp1a1*	Mm 00487218_m1
Tumor necrosis factor	*Tnf*	Mm 00443258_m1
Interleukin 1 beta	*Il1b*	Mm 01336189_m1
Interleukin 6	*Il6*	Mm 99999064_m1
Prostaglandin synthase 2	*Ptgs2* (*Cox2*)	Mm 00478374_m1
Tight junction protein 1	*Zo1*	Mm 00493699_m1
Forkhead box O4	*FoxO4*	Mm 00840140_m1
Interleukin 10	*Il10*	Mm 00439614_m1
